# Suspected duloxetine-induced restless legs syndrome phenotypic variant: a case report

**DOI:** 10.1186/s12888-024-05763-7

**Published:** 2024-05-10

**Authors:** Yan Shao, Yi Chen, Shichang Wang, Chaowei Li, Hongqiang Sun, Xinyu Sun

**Affiliations:** 1 Peking University Sixth Hospital, Peking University Institute of Mental Health, NHC Key Laboratory of Mental Health (Peking University), National Clinical Research Center for Mental Disorders (Peking University Sixth Hospital), Peking University, 51 Huayuan Bei Road, Haidian District, 100191 Beijing, China; 2 Ordos Fourth People’s Hospital, Ordos, China

**Keywords:** Restless arms syndrome, Duloxetine, Antidepressants, Case report

## Abstract

**Background:**

Restless arms syndrome (RAS) is the most common variant of restless legs syndrome (RLS), which is easy to be ignored in clinical practice due to the lack of specific diagnostic criteria. When effective therapeutic agents induced RAS and symptoms persisted after briefly observation, clinicians will face the challenge of weighing efficacy against side effects.

**Case presentation:**

A 67-year-old woman was admitted to a geriatric psychiatric ward with depression. Upon admission, the escitalopram dose was reduced from 15 mg to 10 mg per day, and the duloxetine dose was increased from 60 mg to 80 mg per day. The next night before bedtime, she developed itching and creeping sensations deep inside bilateral shoulders and arms, with the urge to move, worsening at rest, and alleviation after hammering. The symptoms persisted when escitalopram was discontinued. A history of RLS was confirmed. Treatment with 40 mg of duloxetine and 0.125 mg of pramipexole significantly improved depression, and the paresthesia disappeared, with no recurrence occurring 6 months after discharge.

**Discussion and conclusions:**

This case suggests that psychiatrists should pay attention to RLS variants when increasing doses of duloxetine. Long-term improvement can be achieved through dosage reduction combined with dopaminergic drugs instead of immediate discontinuation.

## Introduction

Restless legs syndrome (RLS), also known as Willis-Ekbom disease, is a sensorimotor sleep-wake disorder characterized by an irresistible urge to move and is usually associated with indescribable discomfort, which appears or exacerbates at rest, occurs mainly in the evening and is partially or totally alleviated after activity. It is estimated that 3% of the worldwide adult population reports RLS, with a higher prevalence among women and elderly individuals [1]. Clinically significant RLS has a high prevalence in psychiatric patients [2]. However, the aetiopathogenesis of RLS remains elusive. It has been proposed that brain iron deficiency might induce dopaminergic dysfunction and hyperglutamatergic states, thus altering the functioning of corticostriatal circuits and triggering arousal systems, leading to sleep disturbances and sensorimotor symptoms in RLS patients [3]. Genetic predispositions and environmental factors such as renal failure, pregnancy, and drug administration (e.g., antidepressants) may also be involved [3]. For severe RLS, pharmacological interventions (e.g., dopaminergic drugs) are recommended [3].

Although the abnormal sensations predominantly involve the legs, more than one-third of patients reported other body parts affected, such as the arms (the most frequent regions), back, abdomen, and buttocks [4]. Symptoms that mainly or solely occur in body parts other than the legs are called RLS variants and occur in less than 10% of patients [4]. Compared to those in RLS patients, sleep disturbances are more prevalent in patients with RLS variants [4]. A close relationship between antipsychotic agents, antidepressants, dopamine therapy and restless arms syndrome (RAS) has been described [5, 6]. RAS can be very bothersome and decrease quality of life. Unfortunately, it is still difficult to identify clinically.

## Case description

**Fig. 1:**
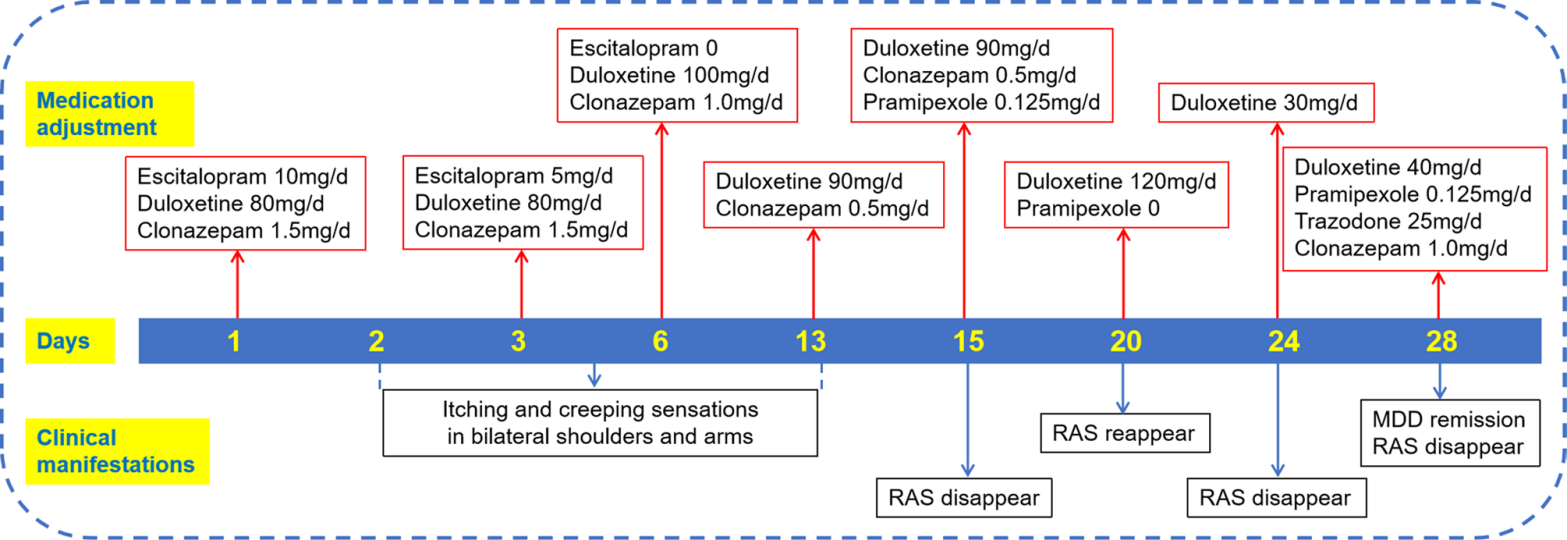
Treatment timeline. MDD, major depressive disorder; RAS, restless arms syndrome

A 67-year-old woman was referred to the geriatric psychiatric ward complaining of depressed mood, anhedonia, nervousness, brain chirping, fatigue, suicidal ideation, decreased appetite and poor sleep starting 5 months after contracting coronavirus disease 2019. She had intermittent depression for more than 31 years, with one to two episodes per year, each lasting about one month. She had regular outpatient visits and took medicine according to the doctor’s instructions. During this period, there were two severe episodes, one in September 2014 due to a long journey, and the other (this episode) in December 2022 after contracting coronavirus disease 2019. The patient responded well to citalopram, escitalopram (maximum daily dose of 20 mg), and duloxetine (maximum daily dose of 60 mg) while intolerant to venlafaxine, bupropion and trazodone. Her mother was diagnosed with mania. The patient had no history of iron deficiency anaemia, hypothyroidism, or other medical conditions and had no history of any psychoactive substance abuse. No obvious abnormalities were found upon physical or neurologic examination or laboratory tests. She was diagnosed with recurrent depressive disorder and received escitalopram 15 mg/d with no obvious effect over 15 weeks of regular administration, followed by 2 weeks of combined use of duloxetine 60 mg/d as an outpatient therapy, which was less effective. Therefore, we reduced the dose of escitalopram to 10 mg/d and increased the dose of duloxetine to 80 mg/d on admission. Clonazepam at an initial dose of 1.5 mg/d was given to promote sleep. On the morning of the third day, the patient reported itching and creeping sensations deep inside her bilateral shoulders and arms before bedtime, with some remission after moving, which had never happened before. A gradual improvement in mood was reached, but a lack of energy persisted. Thus, escitalopram was discontinued, and duloxetine was increased to 100 mg/d on the sixth day. One week later, the patient exhibited continuous itching and creeping, which caused significant disturbances in sleep at night and drowsiness in the morning. Duloxetine was decreased to 90 mg/d, and clonazepam was concomitantly decreased to 0.5 mg/d for the unrelieved paraesthesia. However, the feeling of creep still held. By clarifying the history, we learned that the patient had a history of intermittent leg soreness since age 41, which increased at rest and decreased after stretching, 3–4 times a week, lasting for half an hour to 4 hours each day, and typically occurred in the evening; however, the patient had never been diagnosed with RLS and had not received specific treatment. The total International Restless Legs Study Group Severity Rating Scale score was 25, indicating severe RLS. None of her first-degree family members reported any previous history of RLS. To consolidate the condition, pramipexole 0.125 mg/d was prescribed to relieve the unpleasant sensations, the dose of duloxetine kept the same. The next morning, the patient reported good sleep without any paraesthesia. She experienced a burning stomach after taking pramipexole, which was relieved by vitamin B6, and this sensation disappeared 3 days later. Polysomnography suggested prolonged sleep latency (34.5 min), poor sleep continuity (27 wake times), deep sleep loss (no N3), low sleep efficiency (80.8%), moderate obstructive sleep apnea hypopnea (apnea hypopnea index = 20.3/hour), multiple transient mentalis myoelectric activities in rapid eye movement sleep, and a periodic leg movement index of 3.6/hour during sleep (Table 1 and 2). After five days, an attempt was made to increase the dose of duloxetine to 120 mg/d to improve energy because of insufficient efficacy at a dose of 90 mg/d. Moreover, we stopped pramipexole to determine whether the discomfort worsened. Not surprisingly, she experienced recurrence of unpleasant sensations in her arms. In the next 3 days, the dose of duloxetine was tapered to 30 mg/d, after which the symptoms disappeared. After consultation with the patient, we ultimately prescribed 40 mg/d duloxetine and 0.125 mg/d pramipexole to balance the efficacy and adverse effects (Fig. 1). Since the patient had difficulty falling and maintaining asleep, trazodone 25 mg/d and clonazepam 1.0 mg/d were added. When her mood and sleep stabilized, clonazepam was gradually discontinued after discharge. She reported complete remission of depression and RLS. Over the 6 months of follow-up, the patient comes to the outpatient clinic every month and takes medicine regularly. Her mood remained stable, and no RLS or RAS reappeared. Pramipexole was discontinued to prevent long-term treatment-induced worsening of RLS symptoms. The patient is still undergoing regular outpatient visits.

**Table 1:** Clinical features

Age (years)	67
Sex	Female
Medical conditions	Recurrent depressive disorder
Medications when RAS emerged	Escitalopram 10 mg/d, Duloxetine 80 mg/d, Clonazepam 1.5 mg/d
Psychoactive substance abuse	×
RLS	
Duration (years)	26
Location	Bilateral lower extremities
Abnormal sensation	Sore and swollen
RAS	
Duration	Newly emergent after duloxetine dose increment
Location	Bilateral shoulders and upper limbs
Abnormal sensation	Itching and creeping
Diagnostic criteria	
Urge to move	√
Appearance or exacerbation at rest	√
Relief by movement	√
Night dominance	√
Supportive features	
International Restless Legs Study Group Severity Rating Scale	25
Family history	×
Periodic leg movements of sleep	×
Response to dopaminergic therapy	√
Serum iron (µg/dL)	25
Serum ferritin (ng/ml)	139.7
Serum hemoglobin (g/dL)	133
Disturbed sleep	√

*Abbreviations* RAS, restless arms syndrome; RLS, restless legs syndrome

**Table 2:** Polysomnographic variables

Sleep onset latency (min)	34.5
REM latency (min)	174.5
Total sleep time (min)	417.5
Sleep efficiency (%)	80.8
Time of wake after sleep onset (min)	64.5
Number of wake after sleep onset	27
N1 (%)	13.5
N2 (%)	72.8
N3 (%)	0
REM (%)	13.7
Apnea-hypopnea index (/h)	20.3
Periodic limb movements in sleep index (/h)	3.6
Arousal index (/h)	6.3

*Abbreviations* REM, rapid eye movement

## Discussion and conclusions

Several interesting points about this case are as follows:

1. *The presence of RLS was poorly recognized by physicians.* Symptoms in this patient started in her 40s intermittently and were not diagnosed until her 60s.
2. *The anatomical distributions and abnormal sensory profiles of RLS changed during the course of disease.* The patient presented with bilateral sore legs and was drug naive and then experienced bilateral itching and crawling of the shoulders and upper extremities via the use of antidepressants.
3. *RAS symptoms developed in response to duloxetine in a dose-dependent manner*. Symptoms occurred when the dose of duloxetine was increased from 60 mg to 80 mg, not during the first few days of antidepressant use.
4. *RAS symptoms were relieved with dopaminergic drugs*. The lowest effective dose (40 mg) of duloxetine combined with pramipexole (0.125 mg) maintained a durable balance.

Duloxetine is a potent dual serotonin-norepinephrine and a less potent dopamine reuptake inhibitor approved by the U.S. Food and Drug Administration for the treatment of major depressive disorder [7]. Strong evidence and a high estimated effect size with respect to antidepressant efficacy supported by a Bayesian meta-analysis were found for duloxetine [8]. It is also used to treat general anxiety disorders, diabetic neuropathy, chronic muscle or joint pain, and fibromyalgia. The most common adverse events were nausea, somnolence, dry mouth, hyperhidrosis, constipation, and sedation [9]. A few case reports have described duloxetine-induced RLS [10–12], and the incidence of RLS triggered by duloxetine was less than 5% according to prospective observation [11].

The patient developed an acute onset of unpleasant sensations, described as itching and crawling sensations in her bilateral shoulders and upper arms, associated with an urge to move, predominantly occurring at night; these sensations were partially alleviated after stretching. These findings were consistent with RAS diagnosis, the most common RLS variant. The RAS symptoms were most likely attributable to duloxetine, given that symptom emergence and resolution coincided with the titration (i.e., from 60 mg to 80 mg) and discontinuation of duloxetine. A series of case reports documented dose-dependent induction of RLS by quetiapine [13]; unlike in previous cases, this patient had a history of long, intermittent episodes of RLS (sore legs). During dose escalation of duloxetine (first observed at 80 mg), the symptoms reappeared, and the location (extending from both shoulders to both the upper arms and forearms) and characteristics (itching and crawling) changed.

An important differential diagnosis for RLS is akathisia, a syndrome characterized by subjective complaints of inner restlessness often leading to excessive movements, including pacing back and forth. Akathisia is uncommonly associated with duloxetine (≥ 1/1000 to < 1/100), and it does not worsen specifically at night or be temporarily alleviated through limb movements. Augmentation [6], known to be a side effect of long-term treatment with dopaminergic medications and manifesting as an expansion of symptoms from the legs to other body parts, can be ruled out in this case because the patient had never been treated with dopamine therapy before.

The emergence of RLS during escitalopram therapy should not be ignored. Several case reports have explicitly indicated escitalopram-triggered RLS [11, 14]. However, escitalopram could be ruled out as the cause of RLS in our patient, as her symptoms persisted despite her discontinuing escitalopram. However, pharmacokinetic interactions between escitalopram and duloxetine should be considered. Escitalopram has a weak inhibitory effect on the activity of the cytochrome CYP2D6, while duloxetine is metabolized by CYP1A2 (major) and CYP2D6 (to a lesser extent) [15]. Concomitant administration of escitalopram with duloxetine may theoretically increase the plasma concentrations of duloxetine. This might enhance the synaptic availability of serotonin, which indirectly inhibits dopaminergic neurotransmission.

The association between depression and RLS is clinically relevant. More than 65% of RLS patients had a lifetime history of depression [16]. At least 25% of them had a lifetime history of suicidal ideation or behavior, which was dependent on RLS severity and depression history [16]. Furthermore, RLS occurs five times more commonly among patients with depression than among those without depression [17]. A possible explanation for the link between the two diseases is a shared genetic mechanism. For example, in 5 generations of the Arkansas family, a total of 14 individuals displayed signs and symptoms of Parkinson disease, essential tremor, RLS, or depression, indicating the existence of potential familial genetic factors [18]. In addition, dopamine dysfunction [3, 19], inflammatory factors [20, 21], oxidative stress [22, 23], iron deficiency [3, 24], and pregnancy [25] are common pathophysiologic mechanisms involved.

A shared genetic and pathophysiological basis contributes to a high comorbidity rate of RLS and depression; however, this association is underrecognized by clinicians. Clinicians should be familiar with RLS and routinely inquire about patients if they have a prior and family history of similar symptoms to allow prompt diagnosis and treatment. Antidepressants have the potential to induce or worsen RLS symptoms, further complicating the treatment of depression with comorbid RLS. Based on this report, close attention should be given to atypical RLS symptoms associated with duloxetine, especially as the dosage increases. Observation may be the first choice instead of an immediate medication adjustment [26]. If symptoms persisted, reducing duloxetine to the lowest effective dose and/or adding dopaminergic drugs (e.g., pramipexole) may relieve RLS, striking a balance between efficacy and side effects. Long-term regular follow-up is needed to avoid worsening symptoms caused by long-term use of pramipexole.

## Data Availability

This is a single-patient case report. Data sharing is not applicable to this article as no datasets besides those mentioned in the article were generated or analyzed.

## Abbreviations

MDD: Major depressive disorder
RAS: Restless arms syndrome
REM: Rapid eye movement
RLS: Restless legs syndrome
